# A Novel and Effective Cancer Immunotherapy Mouse Model Using Antigen-Specific B Cells Selected In Vitro

**DOI:** 10.1371/journal.pone.0092732

**Published:** 2014-03-19

**Authors:** Tatsuya Moutai, Hideyuki Yamana, Takuya Nojima, Daisuke Kitamura

**Affiliations:** Division of Molecular Biology, Research Institute for Biomedical Sciences (RIBS), Tokyo University of Science, Noda, Chiba, Japan; Fujita Health University, School of Medicine, Japan

## Abstract

Immunotherapies such as adoptive transfer of T cells or natural killer cells, or monoclonal antibody (MoAb) treatment have recently been recognized as effective means to treat cancer patients. However, adoptive transfer of B cells or plasma cells producing tumor-specific antibodies has not been applied as a therapy because long-term culture and selective expansion of antigen-specific B cells has been technically very difficult. Here, we describe a novel cancer immunotherapy that uses B-cell adoptive transfer. We demonstrate that germinal-center-like B cells (iGB cells) induced in vitro from mouse naïve B cells become plasma cells and produce IgG antibodies for more than a month in the bone marrow of non-irradiated recipient mice. When transferred into mice, iGB cells producing antibody against a surrogate tumor antigen suppressed lung metastasis and growth of mouse melanoma cells expressing the same antigen and prolonged survival of the recipients. In addition, we have developed a novel culture system called FAIS to selectively expand antigen-specific iGB cells utilizing the fact that iGB cells are sensitive to Fas-induced cell death unless their antigen receptors are ligated by membrane-bound antigens. The selected iGB cells efficiently suppressed lung metastasis of melanoma cells in the adoptive immunotherapy model. As human blood B cells can be propagated as iGB cells using culture conditions similar to the mouse iGB cell cultures, our data suggest that it will be possible to treat cancer-bearing patients by the adoptive transfer of cancer-antigen-specific iGB cells selected in vitro. This new adoptive immunotherapy should be an alternative to the laborious development of MoAb drugs against cancers for which no effective treatments currently exist.

## Introduction

Immunotherapy has recently become more widely accepted as an effective means to treat cancer patients. The main player in cell-mediated cancer immunotherapy has been cytotoxic T lymphocytes (CTLs) directed against tumor cells, which recognize via their T-cell receptor (TCR) a particular peptide derived from a tumor antigen (Ag) presented by MHC I on the tumor cells. Such T cells from excised tumor tissues or patients’ blood are selectively expanded in vitro on syngeneic Ag presenting cells (APCs) expressing the tumor Ag with cytokines like IL-2 and then transferred back into the patients [1], [2]. Relatively non-specific versions of cellular immunotherapy have also been clinically tested, including those using T cells and NK cells expanded through stimulation with IL-2 and anti-CD3 antibodies (Abs), with/without additional cytokines [3], [4]. Recently, in-vitro expanded dendritic cells (DCs), which are very efficient APC, have also been used to stimulate tumor-Ag-specific CTLs as well as CD4^+^ T cells in vivo [5]–[7]. These therapies based on adoptive cell transfer have thus far not been commonly adopted as an option for cancer therapy since their clinical success has been limited while they require time-consuming laboratory work, including individual cell culture for several weeks in a quality-controlled clean room.

On the other hand, Ab-based immunotherapy has been growing rapidly as a promising cancer immunotherapy. Indeed, more than a dozen monoclonal Abs (MoAbs) are currently approved for the treatment of cancer in humans [8]–[10]. As an anti-cancer drug, MoAbs have tremendous merits as compared to chemotherapy since they target only the cells expressing specific Ags. The biochemical nature and biological features of each isotype of Abs are well known, and so are the mechanisms by which they mediate target cell lysis, namely, Ab-dependent cellular cytotoxicity (ADCC) and complement-dependent cytotoxicity (CDC) [11], [12]. As naturally existing proteins in all individuals, Abs are expected to have fewer side effects and, as such, it is easier to predict their performance as a drug. As compared to the cell-mediated immunotherapies described above, Ab-mediated immunotherapy is simpler to perform if the supply of the MoAb is adequate. However, the MoAb drugs also have drawbacks: they are expensive and their development is still challenging, requiring considerable time and cost, from animal immunization, through screening of hybridomas, to gene cloning and recombination methods for their humanization, which is necessary to avoid an immune response by the recipient [10], [13]. Tumor Ags that MoAb drugs target are typically transmembrane proteins, which are often difficult to prepare as a soluble immunogen. Moreover, even with humanized MoAbs, residual mouse-derived segments of the V-region can be antigenic in humans and induce human anti-mouse Abs [14]. Because of these issues, pharmaceutical companies tend to limit MoAb targets to those expressed by relatively common cancers.

Given the aforementioned merits of MoAb drugs and the merits of adoptive cell transfer therapies as being primarily custom-made and costing less to develop, it seems plausible to develop a therapy to transfer patient-derived plasma cells that produce tumor-Ag-specific, completely human Ab. However, we are unaware of any case where such a therapy has been successful. Plasma cells are terminally differentiated cells and thus are unable to grow in culture. Instead, B cells, a direct precursor of plasma cells, could be used for the transfer. However, even B cells have proven difficult to expand in a sufficient number for adoptive transfer therapy. In addition, it has not been established whether and to what extent the transferred B cells can survive and differentiate into plasma cells in vivo.

Usually, MoAbs are derived from Ag-specific hybridomas, hybrid cells between splenic B cells from repeatedly immunized animals and a fusion partner plasmacytoma cell line. In the animal immunized with a given Ag, Ag-bound B cells are activated and proliferate to form germinal centers (GCs) in the spleens or lymph nodes. In the GCs, the B cells undergo isotype switching and somatic hypermutation of immunoglobulin genes to increase affinity of their Ag receptors (B-cell receptor, BCR). Among them, the B cells expressing BCR specific to the immunized Ag are selectively expanded and differentiated into memory B cells or long-lived plasma cells (LLPCs) [15], [16]. Upon a final booster immunization, the Ag-specific memory B cells are activated and proliferate to become plasmablasts, which usually form the Ag-specific hybridomas. Thus, although Ag-specific memory B cells can be found in a considerable number in immunized individuals, antigen-specific B cells are usually rare in non-immunized individuals. Therefore, any B-cell adoptive transfer therapy would require a method to selectively expand the rare tumor-Ag-specific B cells from the extremely polyclonal peripheral B cells of the patients.

To develop a system to selectively expand tumor-Ag-specific B cells for adoptive transfer therapy, we utilized the induced GC B (iGB) cell culture system that we recently reported [17]. In this system, mouse naïve B cells are cultured successively with IL-4 and IL-21 on a feeder cell line expressing CD40L and BAFF (40 LB), resulting in the extensive proliferation (up to 10,000 fold in 8 days) of class-switched B cells with a GC phenotype, termed iGB cells. After culture with IL-21 and transfer into irradiated mice, the iGB cells differentiate into plasma cells and tend to colonize the bone marrow (BM) and secrete Abs [17]. By adapting this system to human B cells, it would be possible to prepare large numbers of human B cells that would produce completely human Abs when transferred into patients. Toward our goal of establishing B-cell-mediated adoptive transfer therapy for cancer, we have evaluated in a mouse model how much and for how long the transferred iGB cells produce Ab in non-irradiated mice, and whether they inhibit growth of cancer cells that express an Ag recognized by the same Ab in vivo. In addition, by applying the iGB culture technique, we have developed a system to select relatively rare B cells that bind to a membrane-bound Ag, and showed that the selected B cells are effective in the adoptive transfer cancer immunotherapy model.

## Results

### iGB Cells Colonize the Bone Marrow and Produce Ab after Transfer into Non-irradiated Mice

**Figure 1:**
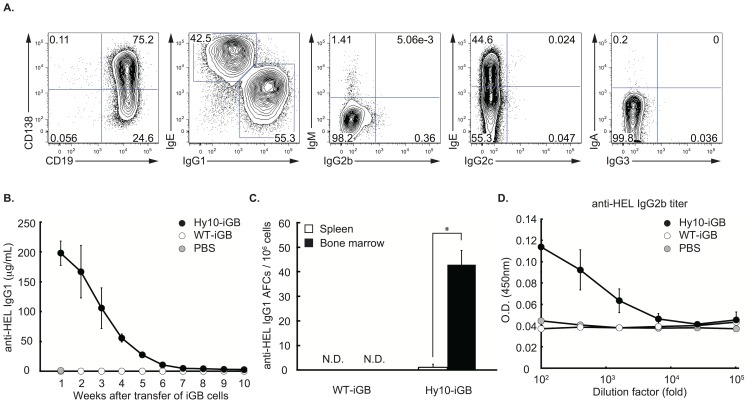
Evaluation of the capacity of iGB cells to differentiate and produce Ab in non-irradiated mice. (A) Splenic B cells from C57BL/6 mice were cultured for 4 days with IL-4, then for 4 days with IL-21 on 40 LB cells. The expression of Ig isotypes, CD19 and CD138 on the expanded iGB cells was analyzed by flow cytometry. Data represent the cells within the lymphocyte gate defined by side- and forward scatter. Numbers indicate the percentages of the iGB cells in the quadrants or windows. Data shown are representative of three independent experiments. (B) Naïve, HEL-binding or total B cells from the spleens of Hy10 or WT C57BL/6 mice, respectively, were cultured as in (A). After the culture, iGB cells were harvested and CD138^−^ IgE^−^ iGB cells (2×10^7^ cells/mouse) were purified and transferred i.v. into non-irradiated C57BL/6 mice. PBS was also injected as a control. These mice were bled at the indicated weeks after transfer and the serum anti-HEL IgG1 concentration was determined by ELISA. The data are expressed as the mean ± S.D. of individual serum of mice (n = 5 for each group). Data is representative of two independent experiments. (C) HEL-binding or total B cells were cultured and transferred into non-irradiated mice as in (B). Four weeks after the transfer, the numbers of AFCs secreting HEL-binding IgG1 among spleen or bone-marrow cells were determined by ELISPOT assay. The mean number ± S.D. of the AFCs in 10^6^ spleen or BM cells is indicated by each bar. Shown are collective data from three independent experiments, each using 3 recipient mice per group. *p<0.001. N.D.: not detected. (D) Anti-HEL IgG2b titers in the serum samples (10-fold dilutions) obtained at 1 week in (B) were determined by ELISA. Each value is the mean ± S.D. of the samples (n = 5 per each group). Data are representative of two independent experiments.

As we reported previously, most iGB cells after the secondary culture with IL-21 have undergone class switching and express either IgG1 or IgE by day 8. Very few of them express IgM, IgG2b or IgA, and almost none express IgG2c or IgG3 (Figure 1A). We showed previously that the iGB cells differentiate to plasma cells in the bone marrow (BM) when they were transferred into irradiated mice. Here we evaluated the Ab production from the iGB-derived plasma cells in non-irradiated mice. The iGB cells were generated from Hy10 mice, which carry a hen egg lysozyme (HEL)-specific heavy chain (VDJ9) and light chain (κ5) genes in knock-in and transgenic configurations, respectively [18]. Among the iGB cells, IgE^−^ CD138^−^ HEL-binding (HEL^+^) cells were FACS-purified and transferred into non-irradiated C57BL/6 (B6) mice, which were bled weekly to measure the concentration of anti-HEL IgG1. As shown in Figure 1B, a high level of HEL-specific IgG1 was detected in the sera a week after the transfer, and then it gradually declined to a low but still detectable level (>1 μg/ml) by 10 weeks. Anti-HEL IgG1 was undetectable in the sera of the control mice that received iGB cells derived from WT B6 mice. Significant numbers of anti-HEL IgG1 Ab-producing cells (APCs) were detected in the BM, but very few in the spleen, of mice that received the Hy10-derived iGB cells 4 weeks previously (Figure 1C). Anti-HEL Ab of IgG2b class, but not of IgG2c or IgG3 (data not shown), was also detectable a week after transfer with Hy10-derived iGB cells but not with WT iGB cells (Figure 1D). Although the exact concentration of the IgG2b anti-HEL could not be estimated because of the lack of a standard isotype-matched anti-HEL Ab, the IgG2b titer was far lower than that of anti-HEL IgG1 (data not shown). Taken together, these data indicate that in-vitro generated iGB cells are able to differentiate into plasma cells that colonize the BM of non-irradiated mice and can continue to produce Ab there for at least 4 weeks.

### iGB Cells Inhibit Lung Metastasis of Mouse Melanoma Cells in vivo

**Figure 2:**
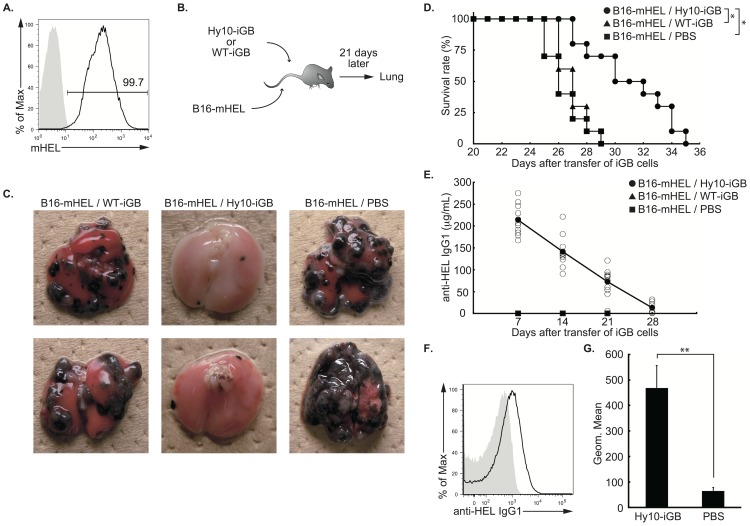
HEL-specific iGB cells inhibit lung metastasis of B16 melanoma cells expressing HEL in mice. (A) Expression of HEL Ag on the B16 melanoma cells transfected with an mHEL expression vector (B16-mHEL). B16-mHEL cells were stained with anti-HEL IgG1 MoAb (black line) or isotype-matched control MoAb (shaded), followed by APC-conjugated anti-mouse IgG1 Ab, and analyzed by flow cytometry. The number indicates the percentage of mHEL-expressing cells. Data is a representative of two independent experiments. (B) Experimental strategy. IgE^−^ CD138^−^ iGB cells (2×10^7^ cells/mouse) derived from HEL-binding splenic B cells of Hy10 mice (Hy10-iGB) or total splenic B cells of WT C57BL/6 mice (WT-iGB), or PBS alone were transferred i.v. into non-irradiated C57BL/6 mice, which had been transferred i.v. with B16-mHEL (C, D, E) or B16-mHEL-GFP (F, G) cells (2×10^5^ cells/mouse) 24 hours before. (C) Photographs of the lungs of the recipient mice described in (B) 3 weeks after the transfer. Images of two mice randomly selected from ten per group are shown. When possible, lungs of the rest of the mice were visually inspected on the day of death. In the non-treated groups, there was fusion of individual metastases into very large tumor masses, making it meaningless to count the number of tumors. (D) Survival rate of the same set of mouse groups (n = 10 per group) as in (B) was compared using LogRank test. *p<0.001. (E) Concentration of serum anti-HEL IgG1 in the same mice used in (D) was determined by ELISA at the indicated time points. Open and closed symbols indicate the values of individual samples and averages of each group, respectively. Data in (D) and (E) are representative of four similar experiments. (F) Binding of anti-HEL IgG1 to B16-mHEL cells in the lung of tumor bearing mice. Lungs of mice that had received Hy10 iGB cells (black line) or PBS (shaded) and B16-mHEL-GFP cells (2×10^5^ cells/mouse) as in (B) were excised 3 weeks after the transfer. Single cell suspensions from the lungs were stained with anti-mouse IgG1-APC and analyzed by FACSCantoII. Representative histograms of the samples gated on GFP^+^ cells are shown. (G) Summary of the experiments shown in (F). Bars represent averages ± S.D. of geometric means (Geom. Mean) of APC fluorescence intensity of the GFP^+^ cells from mice of each group (n = 3). Data are representative of two independent experiments. **p<0.05.

These results suggest a possible application of the iGB cell culture system to clinical use, namely in Ab-mediated cancer therapy. We tested this possibility with a well-studied mouse model of tumor metastasis using the B16 mouse melanoma cell line. We used B16 cells with a membrane-anchored form of HEL (mHEL) [19] as a surrogate tumor Ag, and generated a transfectant clone with homogeneous HEL expression on the cell surface, termed B16-mHEL (Figure 2A). We tested whether HEL-specific iGB cells could inhibit metastasis and growth of the B16-mHEL cells in vivo by producing anti-HEL Abs. Since the HEL-binding affinity of the Hy10 spleen B cells is known to be heterogeneous [18], we sorted those strongly binding HEL from Hy10 spleen B cells and cultured them on 40 LB feeder cells for 3 days with IL-4 and subsequently for 3 days with IL-21 to make iGB cells. Spleen B cells from WT B6 mice were also cultured in parallel. IgE^−^ CD138^−^ B cells sorted from the Hy10 iGB (Hy10-iGB) or WT iGB (WT-iGB) cells, or PBS only as a control, were then injected i.v. into non-irradiated B6 mice that had received B16-mHEL 24 h before (Figure 2B). Lungs of the recipient mice were inspected 3 weeks later. The lungs of the mice that received WT iGB cells or PBS only, had numerous clumps of widely disseminated tumor cells, mostly fusing with each other to form indistinguishable masses. By contrast, only a few small clumps of tumor cells were found in mice that had received Hy10 iGB cells (Figure 2C). As a control, mice inoculated with parental B16 cells developed numerous lung tumors even when treated with Hy10 iGB cells (data not shown).

Long-term observation of the same set of mice revealed that the mice transferred with Hy10 iGB cells survived significantly longer than those transferred with WT iGB cells or only PBS (Figure 2D). Among these mice, serum anti-HEL IgG1 was detected at relatively high concentration in the early period of the time course only in the mice transferred with Hy10 iGB cells, although the Ab concentration gradually declined (Figure 2E). We could show by flow cytometry that the anti-HEL IgG1 was bound to the B16-mHEL cells taken from lung tumors ex vivo 3 weeks after the transfer of Hy10 iGB cells (Figure 2F and 2G). Collectively, these data indicate that HEL-specific Abs produced by iGB-cell-derived plasma cells directly inhibited colonization and/or growth of B16-mHEL cells in the lung and prolonged survival of the recipient mice. Possible mechanisms for the Ab-mediated tumor suppression and possible causes for the eventual death of the treated mice are discussed below.

### Development of a Culture System to Selectively Expand Ag-specific iGB Cells

**Figure 3:**
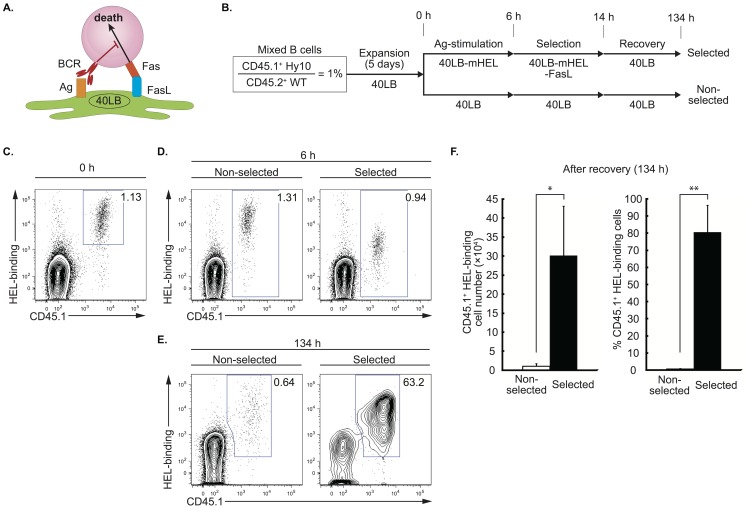
Culture system to selectively expand Ag-specific iGB cells. (A) Schematic representation of the principle of Fas-mediated Ag-specific iGB cell selection (FAIS) system. Only iGB cells whose BCR are ligated with Ag presented on feeder cells become resistant to death via Fas ligation by FasL on the same feeder cells. (B) Protocol for the FAIS system. Splenic B cells from CD45.1^+^ Hy10 mice and CD45.2^+^ WT mice were mixed at a ratio of 1∶99 (1%), and cultured on a 40 LB feeder layer with IL-4 for 3 days and subsequently with IL-21 for 2 days. The resultant iGB cells were step-wise cultured on feeder layers of 40 LB-mHEL for 6 h (Ag-stimulation), 40 LB-mHEL-FasL for 8 h (selection), and 40 LB for 120 h (recovery) in the “selected” protocol. In the “non-selected” protocol, the appropriate number of iGB cells was replated on a feeder layer of 40 LB cells with the same timing as the “selected” protocol. At each time of replating, iGB cells were isolated from the feeder, IgE^+^ and CD138^+^ cells in both protocols. (C–E) Representative flow cytometric profiles (HEL-binding vs. CD45.1; gated on CD19^+^ cells) of the mixed iGB cells before the Ag-stimulation phase (0 h; C), after the Ag-stimulation phase (6 h; D), and after the recovery phase (134 h; E). At each time point, purified iGB cells were stained with biotinylated HEL and streptavidin-APC, anti-CD19 and anti-CD45.1 Abs and analyzed by flow cytometry. The profiles of iGB cells cultured by “selected” (left) or “non-selected” (right) protocol are shown. The numbers in each window represents the percentage of Hy10 iGB cells (CD45.1^+^, HEL-binding) among total CD19^+^ iGB cells. Data are representative of three independent experiments. (F) The absolute number (left) and percentage (right) of the Hy10 iGB cells after the recovery culture with either the non-selected or selected protocol as determined by the analysis shown in (E) are shown as averages ± S.D. of three independent experiments. *p<0.05. **p<0.01.

The results of these in vivo studies suggested that it could be possible to use iGB-cell-mediated tumor therapy in humans. Toward this end, it would be necessary to select presumably rare B cells with specificity for a given tumor Ag. Therefore, we first attempted to develop a model system to enrich and expand Ag-specific mouse B cells present at low levels in the polyclonal B cell pool. We designed a system based on Fas/FasL-mediated apoptosis, since essentially all iGB cells express Fas [17] and are sensitive to Fas-mediated apoptosis (data not shown). In addition, iGB cells become resistant to Fas-mediated apoptosis when their IgG1 BCR is ligated with membrane-bound Ag (data not shown), as previously reported for activated IgM^+^ B cells [20]. Therefore, only Ag-binding iGB cells should survive under conditions where Fas is engaged (Figure 3A). To test this hypothesis, we prepared a model system and generated two new feeder cell lines, 40 LB cells stably expressing a surrogate Ag mHEL (40 LB-mHEL) and those stably expressing mHEL and FasL (40 LB-mHEL-FasL). We initiated the iGB cell cultures on conventional 40 LB feeder cells with a mixture of spleen B cells from CD45.1^+^ Hy10 mice and CD45.2^+^ WT mice at a ratio of 1∶99. After the successive culture with IL-4 and IL-21 on 40 LB cells (expansion), the expanded iGB cells were plated onto 40 LB-mHEL feeder cells and cultured for 6 hours (Ag-stimulation), and then replated on 40 LB-mHEL-FasL for 8 hours (selection), and finally on 40 LB for 5 days (recovery), with IL-21 present throughout after the expansion phase. These specific conditions were determined after many trials with various settings (Figure 3B). After the expansion phase, we confirmed that the proportion of CD45.1^+^ HEL-binding cells remained at 1% (Figure 3C). The proportion remained the same after the Ag-stimulation culture, and did so in the control culture on 40 LB feeder cells as well, although the intensity of HEL staining became lower in the former probably because the BCR was internalized (Figure 3D, “selected”). After the subsequent selection and recovery phases, however, the proportion of CD45.1^+^ HEL-binding cells increased up to ∼80% on average, whereas no enrichment was seen after the parallel control culture on 40 LB cells (“non-selected”). The selected iGB cells mostly expressed BCR of IgG1 isotype (data not shown). Using the “selected” protocol, on average 3×10^5^ HEL-binding B cells were recovered from the culture that began with 10^4^ such cells among 10^6^ B cells in total (Figure 3E and 3F). Thus, we have established a selection culture protocol that enables efficient enrichment and expansion of Ag specific B cells that are present as a small population among a vast majority of non-specific polyclonal B cells. We call this selection system the “Fas-mediated antigen-specific iGB cell selection (FAIS) system”. We have also succeeded in enriching iGB cells specific for the hapten 4-hydroxy-3-nitrophenyl acetyl (NP), initially present at ∼5%, up to ∼80% by essentially the same system using the FasL-expressing 40 LB cells displaying NP-conjugated protein on their surface (data not shown).

**Figure 4:**
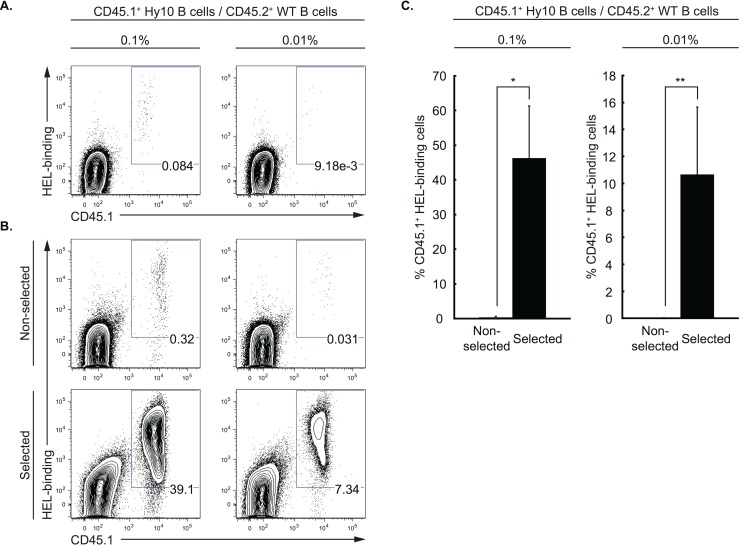
FAIS system can enrich very rare Ag-specific iGB cells. (A and B) Splenic B cells from CD45.1^+^ Hy10 mice were mixed at a frequency of 0.1% or 0.01% with 1×10^6^ CD45.2^+^ WT splenic B cells. The mixed cells (1×10^6^) were cultured as described in Figure 3B. Shown are flow cytometric profiles (HEL-binding vs. CD45.1; gated on CD19^+^ cells) of the mixed cells before the Ag-stimulation phase (0 h; A) and after the recovery phase (134 h; B) in a representative experiment. The number indicated in each window indicates the percentage of Hy10 iGB cells (CD45.1^+^, HEL-binding) among total CD19^+^ iGB cells. (C) The percentage of Hy10 iGB cells after recovery culture in either non-selected or selected protocol initiated from the mixing ratio of 0.1% (left panel, n = 3) or 0.01% (right panel, n = 2), as determined by the analysis shown in (B), are indicated as averages ±S.D. of independent experiments. *p<0.01. **p<0.05.

Next we examined whether fewer Ag-specific B cells in a non-specific pool could be enriched, anticipating the possibility of using this system for clinical application. This time, we started the cultures with CD45.1^+^ Hy10 splenic B cells mixed at a frequency of 0.1 or 0.01% in 1×10^6^ WT B6 splenic B cells (CD45.2^+^), a frequency that was confirmed just before the Ag-stimulation culture of the iGB cells (Figure 4A). Each B-cell mixture was cultured according to the FAIS system (“selected”) or merely on 40 LB cells as a control (“non-selected”). After the recovery culture, the HEL-binding iGB cells were enriched to ∼40% and ∼10% when they were initially present at 0.1% and 0.01%, respectively (Figure 4B and 4C). These data suggest that very rare Ag-specific B cells, as few as 1 in 10^4^, could be enriched and expanded by repeating the FAIS culture protocol.

### In-vitro Selected Ag-specific iGB Cells Suppress Tumor Growth in vivo

**Figure 5:**
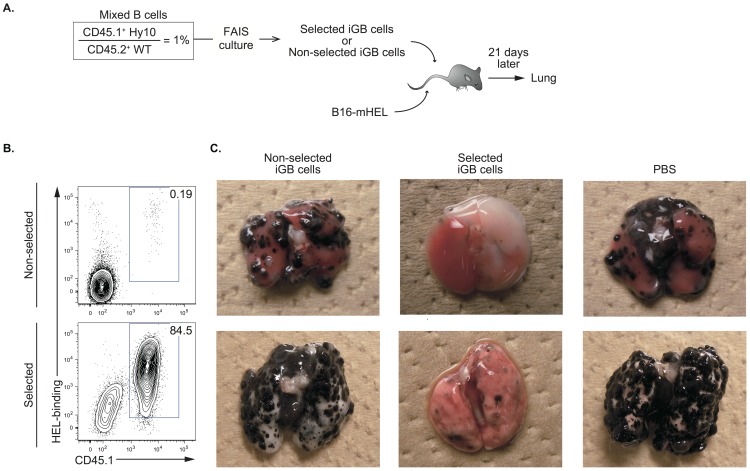
In-vitro selected antigen-specific iGB cells suppress tumor growth in vivo. (A) Experimental strategy. A 1∶99 mixture of splenic B cells from CD45.1^+^ Hy10 mice and CD45.2^+^ WT mice was subjected to the FAIS system as described in Figure 3B. The selected or non-selected iGB cells after the recovery phase, or PBS alone, were injected into non-irradiated C57BL/6 mice that had been transferred i.v. with B16-mHEL cells, as described in Figure 2B. (B) Representative flow cytometric profiles (HEL-binding vs. CD45.1) of iGB cells after the recovery phase of the “selected” and “non-selected” protocols. The numbers in each window indicate the percentage of the Hy10 iGB cells (CD45.1^+^, HEL-binding; gated on CD19^+^ cells) among total CD19^+^ iGB cells. (C) Photographs of the lungs of the mice treated as in (A) 3 weeks after the transfer. Representative images of two mice out of three are shown.

Finally, we tested whether the in-vitro selected iGB cells are an effective anti-tumor therapy in the melanoma metastasis model in mice. CD45.1^+^ HEL-binding B cells from Hy10 mice were mixed with CD45.2^+^ polyclonal B cells from WT B6 mice at a ratio of 1∶99 and cultured in the FAIS system or on 40 LB cells as a non-selected control, as described in Figure 3 (Figure 5A). After the recovery culture, the frequency of the HEL-binding iGB cells reached 85%, a more than 400-fold enrichment, after the FAIS culture compared to in the control culture (Figure 5B). We transferred these iGB cells (2×10^7^) either selected or non-selected, or only PBS, into non-irradiated B6 mice that had been transferred with 2×10^5^ B16-mHEL cells. Three weeks later, B16-mHEL cells were disseminated throughout the lungs and formed numerous clumps of various sizes in the mice that had received non-selected iGB cells or PBS. By contrast, only a small number of tumors, mostly small in size, were observed in lungs of the mice that had received the selected iGB cells (Figure 5C). These data indicate that iGB cells selected in vitro based on their Ag binding specificity are still capable of differentiating into plasma cells in vivo and inhibiting growth of tumor cells that express the same Ag.

## Discussion

Based on results using our mouse model, here we propose a new system of adoptive transfer cancer immunotherapy using B cells. With this system, one can expand naïve B cells to produce a large number of GC-like B (iGB) cells and from them, infrequent Ag-specific B cells can be selected and further expanded by the FAIS system for use in adoptive transfer therapy. We showed that the transferred iGB cells colonized the bone marrow and produced Ab, mainly of the IgG1 class, for several weeks. Using this system, we showed an example of an effective cancer treatment. The transfer of iGB cells specific for a surrogate tumor Ag (HEL) suppressed metastasis and growth in the lungs of melanoma cells expressing the same Ag and prolonged the survival of the recipient mice. If this system can be adapted to work with human B cells, the B-cell adoptive transfer should be a very attractive alternative to MoAb in cancer immunotherapy: it will require a shorter period of time from the identification of a tumor Ag to launch the treatment of patients than producing a humanized MoAb, therefore will serve as a custom-made therapy that could target diseases of low incidence. In addition, human-derived iGB cells should produce complete human Ab in the recipient.

In the present study, it remains to be formally demonstrated how the transfer of iGB cells resulted in the suppression of melanoma growth in the lungs. Considering the high serum titer of the HEL-specific IgG1 sustained at least 4 weeks after the transfer (Figures 1B and 2E) and the binding of such IgG1 to the HEL-expressing melanoma cells ex vivo (Figure 2F and 2G), the tumor suppression is likely to be mediated by the anti-HEL IgG1 produced by the iGB-cell-derived plasma cells. Thus, the mechanisms responsible for the tumor suppression may be ADCC and/or CDC, the same mechanisms ascribed to MoAb drugs in vivo [9], [10]. In this regard, previous studies comparing various isotypes of mouse MoAbs for their anti-tumor effects in vivo as well as in vitro demonstrated that IgG1 showed moderate effects in vivo and in ADCC, but not in CDC, whereas IgG2a was the most effective in most cases, with IgG2b and IgG3 being variable among the reports using different sets of MoAbs and target cells [21]–[24]. Thus, the propensity of the B cells derived from our mouse iGB cell culture system to switch almost exclusively to either IgG1 or IgE isotypes may have limited the efficacy of the therapy in our mouse model; all the mice, even those treated with the Ag-specific iGB cells, eventually died. It should be noted that such mice died with huge clumps of melanoma tumors in the peritoneal cavity, but only a few small tumors were found in their lungs even at death (data not shown), indicating that the anti-tumor activity of Ab isotypes may differ depending on the tissues being infiltrated. Considering a future application for humans, feasibility of iGB cells would depend on the IgG subtype to which human iGB cells would switch in the iGB cell culture.

The success of adoptive B-cell transfer immunotherapy for cancer will depend on how efficiently the tumor-Ag-specific iGB cells can be selected and propagated. For this, we developed a new system to select and expand rare Ag-specific B cells in vitro, termed the FAIS system. This system is based on the characteristics of the iGB cells: first, iGB cells grow enormously and robustly on a 40 LB feeder layer; second, essentially all of them express Fas receptor and die when plated on 40 LB feeder cells expressing FasL; third, they become resistant to the Fas-signal when their BCR is pre-stimulated by a cognate Ag bound on the feeder cells. This system is so simple that, once cancer-specific Ag is identified that can be expressed on the feeder cells, it would be easily testable if B cells binding the Ag can be obtained. Our preliminary data show that human blood B cells can grow and express Fas in a similar culture condition, suggesting that the FAIS system may be applied to human B cells. It remains to be determined how high the affinity of BCR is required for the B cells to be selected out by this system. If the B cells with moderate affinity would be selected, the FAIS system would have to be improved to enable a controllable V gene mutagenesis by AID and repeatable selection-expansion cycles, in order to obtain B cells with a BCR whose affinity to Ag is high enough for clinical applications.

The Ag-specific B cells selected by the FAIS system could also serve as a source of complete human MoAbs, which would be more desirable than “humanized” murine MoAbs, which have mouse/human hybrid V regions, possibly lessening their original affinity to Ags, and making them more immunogenic to humans than the fully human MoAbs [13]. In addition, our system requires less time, cost, and technical skills compared to the conventional methods such as the “humanized” MoAbs, phage display technologies [25], [26] or the lymphocyte microwell-array system [27]–[29]. Phage display technology depends on the quality of the Ab cDNA libraries, which consist of a huge number of random combinations of H and L chains. The lymphocyte microwell-array system requires special devices to detect single cells that emit faint fluorescence. Recently, Spits, Beaumont and colleagues have reported a system to efficiently expand human B cells in vitro and generate human MoAbs from them. They immortalized blood memory B cells by expressing conditionally active STAT5 or Bcl-6/Bcl-xL and cultured the cells with IL-21 on feeder cells expressing CD40 L. From the expanded cells, those binding to viral or bacterial Ags were selected by fluorescence activated cell sorting or limiting dilution methods [30]–[32]. They used B cells from humans or “humanized” mice previously infected or immunized with such pathogens, in which the frequency of Ag-specific memory B cells may be relatively high. It is unknown whether the same system can be applied for selecting presumably rare B cells specific for tumor Ags from unimmunized individuals. Based on the results shown here, our FAIS system may be able to enrich Ag-specific B cells that are as rare as 0.01% in a non-specific B-cell pool, and possibly even less if repeated selection procedures are possible. In addition, our system does not require purified Ags; it is only necessary to express Ags on the feeder cell line by gene transduction. This is advantageous over the other methods described above since most of the target Ags for MoAb immunotherapies are transmembrane proteins that are often difficult to prepare as soluble Ags. Another advantage over the methods requiring in-vivo immunization would be that the in-vitro system is free of T-cell-mediated self-tolerance and therefore may allow the expansion of B cell clones that react with self tumor Ags.

## Materials and Methods

### Ethics Statement

All mouse procedures were performed in accordance with the regulations of the Tokyo University of Science on animal care and use, under the protocols approved by the Animal Care and Use Committee of the Tokyo University of Science (approved protocol #S13009). In the survival study, the tumor-recipient mice were checked daily in the mornings and evenings, and we euthanized the mice when mice could not move owing to the tumor before sacrificing them.

### Mice

C57BL/6 mice were purchased from Japan SLC. Hy10 (formerly called HyHEL10) mice carrying a HEL-specific V_H_ knock-in (VDJ9 ki) allele and an Ig-κ transgene (κ5 tg) [18], [33] were backcrossed to the congenic C57BL/6-CD45.1 strains. Mice 8–10 weeks of age were used for experiments unless indicated otherwise. All mice were maintained in our mouse facility under specific pathogen-free conditions. When we dissected the mice, mice were killed by cervical dislocation under anesthesia with Isoflurane in all mouse experiments.

### Plasmid Construction and Retroviral Transduction

A cDNA encoding a membrane-bound form of HEL (mHEL) excised from pcDNA3-mHEL (a gift of Dr. R. Brink [19]) was inserted into pMX-IRES-GFP [34] to make pMX-mHEL-IRES-GFP. An shRNA sequence targeting the Fas 3′UT sequence, 5′-gtgttctctttgccagcaaat-3′, was inserted into pSIREN-RetroQ vector (Clontech), to make a retroviral vector pSIREN-RetroQ-shFas. An eGFP sequence in the pMX-IRES-GFP vector was replaced with a cDNA consisting of extracellular and transmembrane domains of human CD8 (hCD8) to make pMX-IRES-hCD8. A FasL cDNA was inserted into the pMX-IRES-hCD8 to make pMX-FasL-IRES-hCD8. The retroviral vectors were transfected into packaging cells, PLAT-E [34], using FuGENE (Roche). On the next day, the supernatants were added to target cells in the presence of 10 μg/mL DOTAP Liposomal Transfection Reagent (Roche).

### Cell Lines

B16 mouse melanoma cells [35] were transfected with the pcDNA3-mHEL plasmid by lipofection using Trans IT-LT1 (Takara), and cultured with G418 (2 mg/ml, Wako). Drug-resistant stable clones (B16-mHEL) were subsequently selected. B16 cells were retrovirally transduced with the pMX-mHEL-IRES-GFP, and then cloned by limiting dilution method to establish B16-mHEL-GFP cells. 40 LB, Balb/c 3T3 fibroblasts expressing exogenous CD40-ligand and BAFF, have been described previously [17]. 40 LB cells were transduced with the pMX-mHEL-IRES-GFP vector, and a single clone expressing mHEL and eGFP, termed 40 LB-mHEL, was selected by limiting dilution. To express FasL, 40 LB cells were first transduced with the pSIREN-RetroQ-shFas vector. The resultant Fas-knocked-down cells (40 LB-Fas^−^) were then transduced with the pMX-FasL-IRES-hCD8 vector and a single clone expressing FasL and hCD8 (40 LB-FasL cells) was selected by limiting dilution. Finally, the 40 LB-FasL cells were transduced with the pMX-mHEL-IRES-GFP vector to obtain a single clone expressing mHEL and eGFP (40 LB-mHEL-FasL). B16 and 40 LB cells, and their derivatives, were maintained in D-MEM medium (high glucose; Wako) supplemented with 10% FBS, 100 units/ml penicillin, and 100 μg/ml streptomycin (GIBCO) in a humidified atmosphere at 37°C with 5% CO_2_.

### Isolation of Cells

Naïve B cells were purified from the spleens of the mice mentioned above (“Mice”) by 2-step negative sorting, first by an iMag system (BD Biosciences) using biotinylated MoAbs against CD43 (S7: BD Pharmingen), CD4, CD8α, CD11b, CD49b (DX5), Ter-119 (BioLegend), and streptavidin-particle-DM (BD Biosciences) and then by passing of the unbound cells through a MACS LS column (Miltenyi Biotec), yielding B cells of >97% purity. B cells strongly binding HEL were purified from naïve B cells of Hy10 mice prepared as above by sorting the cells brightly stained with biotinylated-HEL plus streptavidin-APC and with CD19-PE/Cy7 (BioLegend) with FACSAria II (BD Biosciences). HEL (Sigma) was conjugated with biotin using EZ-Link Biotinylation kit (Pierce). iGB cells were purified by removing the feeder cells, IgE^+^ cells and plasmablasts/plasma cells with an iMag system as described previously [17] using primary MoAbs against H-2K^d^ (Biolegend), IgE (R35–72: BD Pharmingen), CD138 (281–2 : BD Pharmingen), and FasL (MFL3 : Biolegend) when removing feeder cells expressing FasL. Purified naïve B cells were cultured on a feeder layer of irradiated 40 LB cells with IL-4 and IL-21, sequentially, to generate iGB cells, as described previously [17]. The purified iGB cells were used for the adoptive transfer into non-irradiated recipient mice, as described below.

### Ag-specific iGB Cell Selection System

The iGB cell culture [17] was performed with the primary culture with IL-4 (1 ng/mL) for 3 days and the secondary culture with IL-21 (10 ng/mL) for 2 days. The following culture was done with IL-21 alone throughout. From the cultured cells, IgE^−^ CD138^−^ iGB cells were purified as described above and seeded onto a feeder layer of 40 LB-mHEL cells (2×10^7^ cells/dish) and cultured for 6 hours. Then the iGB cells were purified again, seeded onto a feeder layer of 40 LB-mHEL-FasL (2×10^7^ cells/dish) and cultured for 8 hours. Finally, surviving iGB cells were purified with an iMag system using MoAbs against H-2K^d^ and FasL and seeded onto a feeder layer of 40 LB cells and cultured for 120 hours. As a control, iGB cells were replated on the feeder layers of 40 LB with the same timing as in the selection protocol.

### Flow Cytometry

Single cell suspensions were treated with anti-CD16/32 Ab to block FcγRII/III before staining as described previously [17], and stained with various combinations of the following Abs: FITC-, PE-, biotin-, PE-Cy7-, allophycocyanin (APC)-, or Brilliant Violet 421™-conjugated Abs against IgM, IgG1, IgG2b, IgG2c, IgG3 (Southern Biotechnology), IgA, IgM, IgE, CD19, CD45.1, CD138 (BioLegend), IgE, and CD138 (BD Pharmingen), or biotinylated HEL. Cells were stained with propidium iodide (PI) just before analysis to eliminate dead cells in the data analyses. When the iGB cells were analyzed, 40 LB feeder cells were gated out based on FSC versus SSC. All samples were analyzed using a FACSCalibur or FACSCanto II (BD Biosciences). The data were analyzed using FlowJo (Tree Star, Inc.).

### Adoptive Transfer of iGB Cells

iGB cells after the secondary culture with IL-21, derived from Hy10 or WT mice of C57BL/6-CD45.1 background, were injected i.v. into non-irradiated C57BL/6-CD45.2 mice (2×10^7^ cells/mouse). HEL-specific Ab forming cells (AFCs) in spleen and BM of the recipient mice were detected by ELISPOT assay 4 weeks after the transfer. HEL-specific Abs in the sera of the recipients were measured by ELISA. ELISPOT and ELISA were performed as described previously [17], [36]. As a cancer therapy model, non-irradiated C57BL/6 mice were transferred i.v. with B16-mHEL or B16-mHEL-GFP cells (2×10^5^ cells/mouse) and, 24 hours later, with iGB cells (2×10^7^ cells/mouse) derived from the Hy10 or WT mice. Survival of the recipient mice was checked daily in the mornings and evenings. Where indicated, lungs of the recipient mice were excised 3 weeks after the tumor transfer and photographed. To examine Ab binding to the tumor cells in vivo, the lungs of the mice transferred with B16-mHEL-GFP and Hy10 iGB cells were excised 3 weeks after the transfer and digested using Collagenase Type1 (GIBCO), and then the single cell suspension was stained with anti-mouse IgG1-APC and analyzed by flow cytometry.

### Statistical Analysis

Statistical analysis was performed using the Student’s t test as appropriate. To assess survival rate, the Kaplan-Mayer model was used and comparison of survival between groups was performed using the LogRank test with XLSTAT software (Addinsoft SARL, Paris, France).
